# Short-Term Deprivation Does Not Influence Monocular or Dichoptic Temporal Synchrony at Low Temporal Frequency

**DOI:** 10.3389/fnins.2020.00402

**Published:** 2020-04-28

**Authors:** Yiya Chen, Seung Hyun Min, Ziyun Cheng, Shijia Chen, Zili Wang, Chunwen Tao, Fan Lu, Jia Qu, Pi-Chun Huang, Robert F. Hess, Jiawei Zhou

**Affiliations:** ^1^State Key Laboratory of Ophthalmology, Optometry and Vision Science, School of Ophthalmology and Optometry and Eye hospital, Wenzhou Medical University, Wenzhou, China; ^2^Department of Ophthalmology and Visual Sciences, McGill Vision Research, McGill University, Montreal, QC, Canada; ^3^Department of Psychology, National Cheng Kung University, Tainan, Taiwan

**Keywords:** monocular deprivation, temporal synchrony, interocular suppression, temporal processing, visual plasticity

## Abstract

Studies on binocular combination and rivalry show that short-term deprivation strengthens the contribution of the deprived eye in binocular vision. However, whether short-term monocular deprivation affects temporal processing *per se* is not clear. To address this issue, we conducted a study to investigate the effect of monocular deprivation on dichoptic temporal synchrony. We tested ten adults with normal vision and patched their dominant eye with an opaque patch for 2.5 h. A temporal synchrony paradigm was used to measure if temporal synchrony thresholds change as a result of monocular pattern deprivation. In this paradigm, we displayed two pairs of Gaussian blobs flickering at 1 Hz with either the same or different phased- temporal modulation. In Experiment 1, we obtained the thresholds for detecting temporal asynchrony under dichoptic viewing configurations. We compared the thresholds for temporal synchrony between before and after monocular deprivation and found no significant changes of the interocular synchrony. In Experiment 2, we measured the monocular thresholds for detecting temporal asynchrony. We also found no significant changes of the monocular synchrony of either the patched eye or the unpatched eye. Our findings suggest that short-term monocular deprivation induced-plasticity does not influence monocular or dichoptic temporal synchrony at low temporal frequency.

## Introduction

Hubel and Wiesel (1963) first demonstrated that visual experience in early life can shift ocular dominance in the feline visual system. For instance, the closure of one eye during the critical period, and hence blocking any form of visual input entering the eye, for a period of days or weeks shifts the eye dominance favoring the non-deprived eye. Monocular deprivation modifies ocular dominance in favor of the non-deprived eye at the expense of the deprived eye. This change was demonstrated both at the functional and structural levels of the ocular dominance columns in V1. They replicated the study in older cats and showed that the adult visual system is not as susceptible to visual experience (Hubel and Wiesel, 1970). This work ushered the belief that neural plasticity peaks immediately after birth and tapers off after the critical period.

Although monocular deprivation can shift the ocular dominance in favor of the non-deprived eye in young animals recent studies of humans have demonstrated that the adult visual system retains some degrees of neural plasticity, albeit of a different form (Levi, 2005; Thompson et al., 2008; Levi and Li, 2009; Lunghi et al., 2011; Clavagnier et al., 2013; Zhou et al., 2013a; Campana et al., 2014). For instance, patching an eye for a brief period of time (from 15 min to 5 h) has been found to shift perceptual ocular dominance in adults *favoring the deprived eye* for only up to 30–90 min (Lunghi et al., 2011; Zhou et al., 2013a; Kim et al., 2017; Min et al., 2018). The shift in perceptual ocular dominance seems to be reciprocal, whereby the deprived eye’s contribution to binocular vision strengthens and that of the non-deprived eye weakens. This reciprocal change in perceptual ocular dominance has been demonstrated with psychophysical methods, such as binocular rivalry and combination (Lunghi et al., 2011; Zhou et al., 2013a), for review see Basgoze et al. (2018). Zhou et al. (2014) and Kim et al. (2017) also showed that this deprivation effect could be induced without completely removing visual input in the deprived eye. Moreover, electrophysiological (Lunghi et al., 2015a; Zhou et al., 2015) and neuroimaging studies (Lunghi et al., 2015b; Chadnova et al., 2017; Binda et al., 2018) have also shown the reciprocal shift. To illustrate, Chadnova et al. (2017) reported an increased response of the deprived eye and decreased response of the non-deprived eye after short-term patching using MEG. They postulated that contralateral inhibition – which is known to regulate the contrast gain of each eye prior to binocular combination in a current model of binocular interaction (Meese et al., 2008) – mediates the patching effect (Chadnova et al., 2017). Using electrophysiology, Lunghi et al. (2015a) found that the amplitude of visually evoked potentials in the deprived eye increased whereas those from the non-deprived eye decreased. The strengthening of the deprived eye after short-term patching has been linked to reduced levels of GABA in the primary visual cortex (Lunghi et al., 2015b).

Short-term deprivation in human adults have also been shown to influence monocular visual functions. For example, Zhou et al. (2013a, c) reported that short-term patching increases the contrast sensitivity of the deprived eye and decreases that of the non-deprived eye in both normal and amblyopic observers. Furthermore, Zhou et al. (2017) reported that the changes in monocular contrast sensitivity for chromatically defined stimuli were similar with that for achromatic- defined stimuli after 2.5-h monocular deprivation. Finally, Binda et al. (2018), using fMRI BOLD responses, showed that monocular deprivation can affect selectivity for spatial frequency in V1. They found that the selectivity for high spatial frequencies was enhanced in the previously deprived eye. Although short-term monocular deprivation affects monocular visual functions, the reciprocal nature of these monocular changes suggest that the patching effect is based on binocular interaction.

Most psychophysical studies have measured the patching effect in the context of spatial vision with behavioral measurements such as binocular combination and rivalry. In a phase combination task, fusible horizontal gratings are shown to different eyes. Measurement of the bias in a fused percept can quantify each eye’s contribution to binocular vision (Ding and Sperling, 2006; Zhou et al., 2013a). Conversely, two incompatible but orthogonal gratings are shown to different eyes in a binocular rivalry task. By measuring the perceived relative duration of each eye’s grating stimulus for each subject, one can quantify the changes in eye dominance after patching (Lunghi et al., 2011). However, different neural mechanisms may be involved in these two psychophysical tasks (Bai et al., 2017; Baldwin and Hess, 2018). For testing monocular functions, gratings with different spatial frequency and different contrast are used to measure the contrast threshold (Zhou et al., 2013a). Although these psychophysical studies have demonstrated the spatial influence of short-term monocular visual deprivation in human adults, they have not shown whether patching influences temporal processing.

One aspect of temporal processing of visual information in the human relates to when an observer determines whether two stimuli are temporally synchronous. Temporal synchrony reflects the dynamic nature of visual processing. It has been shown to be an effective cue for binding and segmenting different signals in the absence of spatial cues (Rideaux et al., 2016). Temporal synchrony threshold, the minimum degree of temporal phase difference that enables observers to determine whether the target is flickering asynchronously in time, has been measured in the normal population (Hess and Maehara, 2011). It has also been used to assess temporal deficits in patients with amblyopia (Huang et al., 2012). Huang et al. (2012) reported that the temporal synchrony threshold of the amblyopic eye is higher than that of the fellow eye. They proposed that temporal processing deficit, rather than the detectability of the target, increases the temporal synchrony threshold in the amblyopic eye. Moreover, Tao et al. (2019) found that the elevation of temporal synchrony threshold in amblyopia was present not only when stimuli was presented to the amblyopic eye (i.e., monocular temporal synchrony) but also when presented dichoptically to amblyopic and fellow eyes (i.e., dichoptic temporal synchrony; or, interocular temporal delay). These findings suggest that there is clinical relevance to studying temporal processing in the human visual system.

In this study, we investigated whether short-term monocular deprivation could influence temporal processing of visual information, namely, the threshold for detecting temporal synchrony. A similar temporal synchrony paradigm was used as the one in the study of Tao et al. (2019). The patching effect was quantified by comparing the threshold for temporal synchrony before and after 2.5 h of monocular opaque patching. Specifically, thresholds for detecting dichoptic temporal asynchrony under dichoptic and monocular viewing were measured. Our results show that monocular deprivation does not influence either monocular or dichoptic temporal synchrony.

## Materials and Methods

### Participants

Ten subjects (23 ± 0.42 years old; four males) with normal or corrected-to-normal vision (logMAR ≤ 0.0) participated in this study. All subjects were naive to the purpose of the study.

### Apparatus

We performed our experiments with a Macintosh laptop equipped with Matlab (Mathworks, Natick, MA, United States) and the Psychtoolbox 3.0.14. We dichoptically displayed the stimuli on gamma-corrected head-mounted 3D goggles (Goovis Pro, NED Optics, Shenzhen, China). The OLED goggles had a resolution of 1600 × 900 pixels (corresponding to 46 × 26 degrees) and a refresh rate of 60 Hz in each eye. The maximal luminance of the OLED goggles was 150 cd/m^2^.

The temporal response functions (TRFs) of the OLED monitor and Cathode Ray Tube (CRT) monitor are not the same. To address whether the TRF of the used display would confound our experimental results, we used Ito et al. (2013)’s measures of the TRFs for the CRT and OLED monitor (see Supplementary Figures S1A,B in the supplementary) to simulate the display outputs and investigate whether the asynchrony signal in our test (i.e., the temporal lag) was varied across different TRFs. Two temporal profiles with a flickering rate of 1 Hz and 100 ms temporal lag were used in this simulation (Supplementary Figure S1C). The temporal profiles of the stimuli (Supplementary Figure S1C) were convolved with the TRFs for the CRT and OLED monitor (Supplementary Figures S1A,B) and the results showed the temporal lag did not change with the tested TRF (Supplementary Figures S1D–F). In summary, the temporal lag threshold measured from our experiment did not confound the TRF of the OLED display. In fact, since our psychophysical task relied on the comparison between two dots, it would work on all the dots simultaneously regardless of which screen was used; it would not selectively affect one dot or dots in one eye. Thus, our measure of synchrony would not be limited by the screen response characteristics.

### Design

**FIGURE 1:**
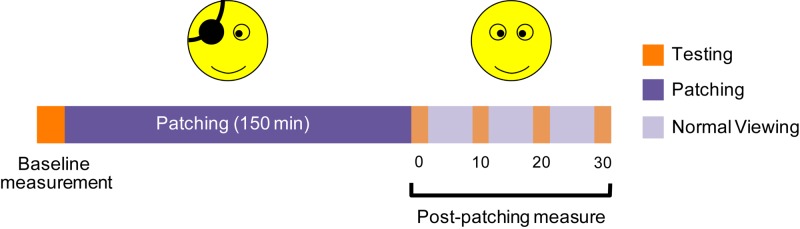
An illustration of the experimental procedure. We deprived one eye for 2.5 h, and assessed the temporal synchrony thresholds at baseline, and 0, 10, 20, 30 min after the finish of the 2.5-h of deprivation.

All subjects participated in two experiments. Each experiment had three stages (Figure 1): baseline measurement of temporal synchrony before deprivation, monocular deprivation for 2.5 h and measurement of temporal synchrony after deprivation. We deprived the dominant eye [tested by the hole-in-the-card test (Dane and Dane, 2004)] for all subjects with an opaque patch (no transmission contrast or luminance). During patching, the participants performed typical office tasks such as browsing a web or reading.

We used a similar paradigm to Tao et al. (2019)’s study to measure the threshold for detecting temporal asynchrony. In this paradigm, two pairs of Gaussian blobs were presented; one pair flickered synchronously (i.e., reference), and the other asynchronously (i.e., signal). They flickered at a temporal frequency of 1 Hz. The reason why we measured at this low temporal frequency was that a higher temporal frequency of the blobs would reduce precise measurement and this also ensured there were no afterimages. For Gaussian blobs with a temporal frequency of 1 Hz (the contrast of blob is modulated sinusoidal over time), one cycle of the stimuli included 60 frames in 1 s since our display screen had a refresh rate of 60 Hz. Therefore, in this case, the minimum measurement accuracy would be 6 degrees (i.e., 360 degrees / 60 frames). On the other hand, at a higher temporal frequency, one cycle of the stimuli would include less than 60 frames, resulting in poorer measurement accuracy. In addition, we performed a pilot study using a higher temporal frequency of the blobs and found that the observers could not perform the task.

We modulated the temporal phase difference between the asynchronously flickering blobs to manipulate the degree of the asynchrony. The two blobs in each pair were presented diagonally with a separation of 2.46 degrees horizontally and vertically. The center of the two blobs was 4.3 degrees above or below the fixation. Between trials, the standard deviation of each Gaussian blob’s size randomly varied from 0.28 to 0.46 degrees, and their luminance contrast varied from 0.4 to 0.8 to prevent participants from using local size or contrast cues to solve the task.

**FIGURE 2:**
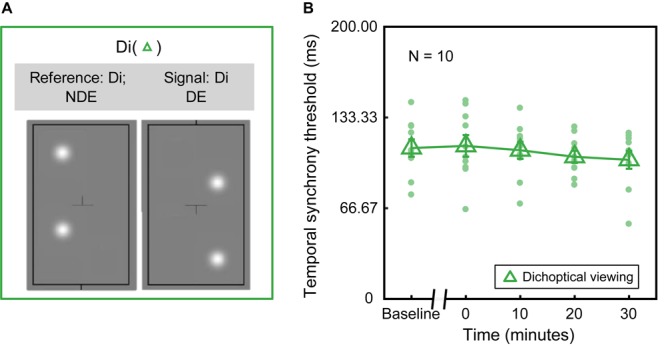
Deprivation effect under dichoptic viewing configuration. **(A)** An illustration of the Di configuration. Both the signal and reference blobs were presented dichoptically to different eyes. **(B)** Dichoptic temporal synchrony threshold in the function of various time points before and after deprivation. Each green dot represents the threshold of each subject. Green triangle denotes the average threshold across ten subjects. Error bars represent standard errors.

**FIGURE 3:**
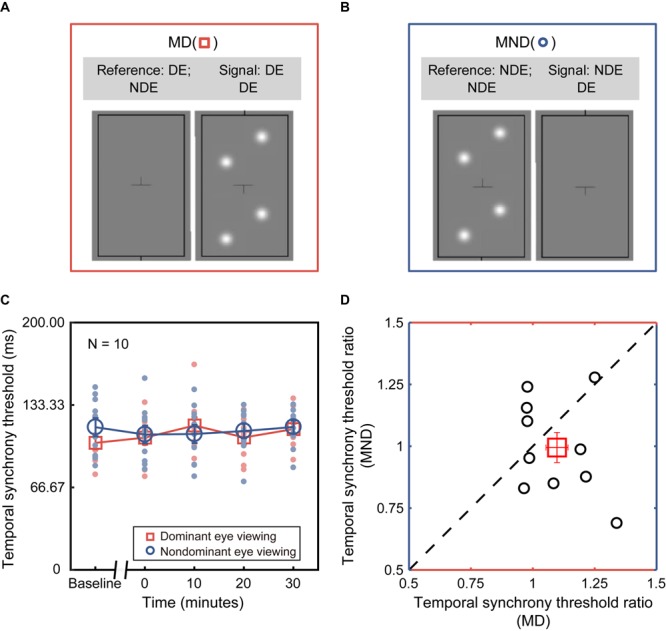
Deprivation effect under monocular viewing configurations. **(A)** Monocular dominant eye viewing (MD). Both signal and reference blobs were presented to the dominant eye (i.e., the assigned patched eye). **(B)** Monocular non-dominant eye viewing (MND). Both signal and reference blobs were presented to the non-dominant eye (i.e., the assigned unpatched eye). **(C)** Monocular temporal synchrony threshold in the function of various time points before and after deprivation. The red plot corresponds to MD configurations, and blue plot to MND. Each dot (blue or red) represents the threshold of each subject. Open symbols (blue circle or red square) denote the average threshold across ten subjects. Error bars (blue or red) represent standard errors. **(D)** Correlation between the changes of monocular temporal synchrony in the deprived eye and non-deprived eye. Error bars represent standard error.

In Experiment 1, we measured the dichoptic temporal synchrony threshold in dichoptic viewing configuration (“Di” as shown in Figure 2A), in which both the signal and reference blobs were presented dichoptically to different eyes. In Experiment 2, we measured the monocular temporal synchrony threshold in two additional monocular viewing configurations, i.e., MD (Figure 3A): monocular dominant eye (i.e., the assigned patched eye) viewing, where both signal and reference blobs were presented to the dominant eye; MND (Figure 3B): monocular non-dominant eye viewing, where both signal and reference blobs were presented to the non-dominant eye. Throughout this paper, we will refer to the three conditions with the abbreviations Di, MD, and MND.

All subjects performed each viewing configuration on a separate day. For each viewing configuration, the temporal synchrony threshold was measured before deprivation and at 0, 10, 20, and 30 min after the 2.5-h of monocular deprivation. An illustration of the experimental procedure is provided in Figure 1. Each test session contained 160 trials (eight temporal phase difference × 20 repetitions) in one measure, which took about 5 min to complete. Before each experiment, subjects were asked to perform at least 160 practice trials.

### Procedure

A constant stimuli method was used to measure the minimum degree of asynchrony that observers needed to discriminate the signal blobs (i.e., the pair of asynchronous blobs). Eight levels of temporal lag (i.e., temporal phase difference between the pair of asynchronous blobs), ranging from 33.33 to 266.67 ms and a step size of 33.33 ms, were tested for each viewing configuration (i.e., Di, MD, and MND). In each trial, the stimuli were presented for 1 s. Participants were asked to determine whether the position of signal blobs was above or below the fixation (two-alternative forced choice, 2AFC). The next trial started 750 ms after the participants’ response. The eight levels of temporal lag were tested using an order randomized in various trials.

### Data Analysis

For each participant, we derived the psychometric function defined as the proportion correct as a function of the temporal lag. The psychometric function of each configuration at each time point was fitted using Palamedes 1.8.1 (Prins and Kingdom, 2018) based on the following equation:

ψ⁢(x;α,β,γ,λ)=γ+(1-γ-λ)⁢F⁢(x;α,β)

(1) =γ+(1-γ-λ)[1-exp(-(x/α))β]

where, F (x; α, β) is the Weibull function; x is the temporal lag; α is the threshold; β is a free parameter related to the slope of the function; γ is the guessed rate; and λ is the lapse rate. During our fitting, we set γ at 0.5 and constrained the λ to a fixed value (ranging from 0 to 0.06) for each fitting. A maximum likelihood method was used for deriving the threshold and slope of the psychometric function for each testing time point of each subject.

## Results

### Experiment 1: Deprivation Effect Under Dichoptic Viewing Configuration (Di)

To assess whether monocular deprivation influences dichoptic temporal synchrony (i.e., the minimum detectable interocular delay), we performed the Di configuration (Figure 2A). The averaged and individual temporal synchrony thresholds as a function of time before and after patching are plotted in Figure 2B. We conducted a Shapiro–Wilks test to check for normality assumption (*p* > 0.05). Then, a one-way repeated-measures ANOVA was used (one within-subject: time of measurements before and after patching) to check whether the changes in the temporal synchrony threshold induced by monocular deprivation was significantly different relative to the one measured in baseline. One-way repeated-measures ANOVA showed that the effect of deprivation on the temporal synchrony threshold was not significant [*F*(4,36) = 1.464, *p* = 0.233]. In other words, no significant difference in the dichoptic temporal synchrony threshold was found between before and after patching.

### Experiment 2: Deprivation Effect Under Monocular Viewing Configurations (MD and MND)

To assess whether monocular deprivation shifts the threshold for temporal synchrony of one eye – be it the dominant (i.e., the assigned patched eye) or non-dominant eye – we obtained temporal synchrony thresholds in both the MD and MND configurations. The averaged and individual temporal synchrony thresholds as a function of time before and after patching are plotted in Figure 3C. One-way repeated-measures ANOVA showed that the effect of deprivation on the temporal synchrony threshold was not significant under either MD [i.e., the assigned patched eye: *F*(4,36) = 0.332, *p* = 0.855] or MND viewing configuration [i.e., the assigned unpatched eye: *F*(4,36) = 2.260, *p* = 0.167]. In short, we found no significant difference in the monocular temporal synchrony threshold before and after patching in both MD and MND configurations.

To further address whether there would be a difference in monocular temporal synchrony threshold between the deprived eye and the non-deprived eye, we conducted an additional two-way repeated-measures ANOVA, with the configuration (two levels) and time point of measurements after patching (four levels) selected as within-subject factors. We found that there was no significant difference between configurations [*F*(1,9) = 1.426, *p* = 0.263] and time points [*F*(3,27) = 0.741, *p* = 0.537]. To better illustrate the relation between the changes of temporal synchrony in deprived eye and non-deprived eye, we divided the value of post-patching tests by the value of baseline to obtain the threshold ratio, and averaged the four post-test ratios. Then we plotted the averaged ratios of non-deprived eye as a function of the average ratios of the deprived eye in Figure 3D. There was no significant difference [paired-*t* test, *t*(9) = 1.194; *p* = 0.263] and no significant correlation (*r* = −0.318, *p* = 0.370) between them.

## Discussion

In this study, we investigated whether short-term monocular deprivation could influence temporal processing between the two eyes using a temporal synchrony paradigm. Our results show that short-term monocular deprivation does not affect the dichoptic temporal synchrony threshold in normal observers.

Previous studies – be they psychophysical, neurophysiological or neuroimaging investigations – have reported that short-term monocular deprivation induces neuroplastic changes in the visual system by shifting the perceptual ocular dominance in favor of the deprived eye (Lunghi et al., 2011; Zhou et al., 2013a). Both translucent (20% luminance reduction) and opaque patches (no light transmission or contrast) have been shown to replicate the patching effect (Zhou et al., 2013a). A contrast-gain control model (Ding and Sperling, 2006) has been proposed to underlie the sensory balance between the eyes (Zhou et al., 2013a, b): During patching, the patched eye’s contrast-gain would be elevated as a consequence of the loss of visual input. Immediately after patch removal, the previously deprived eye would have its contrast-gain restored to baseline values. Due to the reciprocal nature of the interocular inhibitory circuit (Meese et al., 2006), a reciprocal change would occur for the contrast gain of the non-deprived eye. This explanation is supported by both psychophysical (Zhou et al., 2013a) and neurophysiological studies (Lunghi et al., 2015a, b; Zhou et al., 2015; Chadnova et al., 2017). Assuming that changes in contrast-gain control result in changes in the speed of visual processing, we hypothesized that there may be reciprocal changes in the speed of visual processing in the two eyes after a period of monocular deprivation which might translate to elevated thresholds for temporal synchrony. However, our results show that no significant difference exists between the temporal synchrony thresholds before and after patching when stimuli are either dichoptically or monocularly presented.

Temporal synchrony provides an effective cue for integration and segmentation (Rideaux et al., 2016). Segmentation from temporal synchrony has been shown to be achieved by neurons in the early stage of visual processing (Goodbourn and Forte, 2013). An attenuated and delayed hemodynamic response function in early visual cortex (i.e., reduced synchrony of neural firing) due to abnormal interocular suppression, has been proposed as a possible cause for the temporal synchrony deficits in amblyopia (Farivar et al., 2011; Huang et al., 2012; Tao et al., 2019). Therefore, the processing of temporal synchrony occurs primarily in the early visual cortex. Moreover, electrophysiological studies (Lunghi et al., 2015a; Zhou et al., 2015) have suggested that short-term monocular patching can affect early visual areas, especially primary visual cortex (V1). Also Binda et al. (2018) confirmed that the effect of short-term monocular deprivation was most robust in V1, and moderate in V2, V3 and V4 but absent in V3a and hMT+ via fMRI. However, these studies mainly report the changes of response amplitude after patching rather than those of response timing. An unperturbed temporal synchrony threshold may be the result of little to no influence on the synchrony of neural firing by patching. However, there are multiple functional columns in V1 (Daw, 2006). Therefore, despite our findings of no effect on temporal synchrony detection from patching, it would be inappropriate to conclude that patching minimally affects temporal processing.

Another possible factor is that the patching effect on the temporal processing is too small to be detected by our paradigm. Hess and Maehara (2011) reported that we are surprisingly poor at making temporal synchrony judgements, of the order of 30 milliseconds. Therefore, we performed a power analysis based on the variance from our samples (*n* = 10), i.e., σ_*d*_ = 13.414 for Di configuration, σ_*d*_ = 22.054 for MD configuration, σ_*d*_ = 12.835 for MND configuration. To reach a power of 80%, the effect size would need to be *E* = 11.88 ms, *E* = 19.53 ms, and *E* = 11.36 ms for Di, MD, MND configuration, respectively. Thus, any change in temporal processing that occurs at a finer level than this and that impacts other temporal processes would not have been reflected in our approach using temporal synchrony.

Psychophysical studies on short-term monocular deprivation have shown conflicting results. It seems that findings from one task might not agree with those from other tasks because a specific psychophysical task can target distinct level of spatial processing for visual information. Binocular rivalry and combination tasks have shown different results from identical manipulation of visual information. For example, scrambling the phase of a dichoptic movie in one eye has been shown to elicit the patching effect in a binocular rivalry task (Bai et al., 2017) but not in a phase combination task (Zhou et al., 2014). Also, after short-period patching with a translucent patch, the changes in eye dominance were found to be much stronger and longer-lasting for chromatically defined stimuli than achromatically defined ones in binocular rivalry (Lunghi et al., 2013), whereas the changes were similar for the two kinds of stimuli in binocular combination (Zhou et al., 2017). Baldwin and Hess (2018) used two different masks (parallel vs. cross-oriented) to mimic binocular rivalry and combination. Not finding any correlation between the decrease in detection threshold across the two masks, they concluded that short-term monocular deprivation induces multiple separable effects. We suspect that the task-difference of the patching effect that has been reported in the field of spatial vision might also exist in the temporal vision. Here we tested one specific example of temporal processing, namely temporal synchrony. A future study should investigate whether short-term monocular deprivation affects other aspects of temporal processing in the visual system such as single-event asynchrony judgments, unimodal (visual) or cross-modal (e.g., audio-visual).

## Data Availability Statement

All datasets generated for this study are included in the Supplementary Data Sheet S1.

## Ethics Statement

The studies involving human participants were reviewed and approved by Ethics Committee of the Wenzhou Medical University. The participants provided their written informed consent to participate in this study.

## Author Contributions

YC, SM, FL, JQ, P-CH, RH, and JZ conceived the experiments. YC, ZC, SC, ZW, and CT performed the experiments. YC, SM, ZC, and JZ analyzed the data and interpreted the data. YC, SM, P-CH, RH, and JZ wrote the manuscript. All authors contributed to manuscript revision, read and approved the submitted version.

## Conflict of Interest

The authors declare that the research was conducted in the absence of any commercial or financial relationships that could be construed as a potential conflict of interest.

## Supplementary Material

FIGURE S1 The temporal characteristics for the CRT and OLED monitor. **(A,B)** The temporal response function (TRF) for CRT and OLED. **(C)** The temporal profiles of the stimuli. The solid and dashed lines represent the two stimuli. Two stimuli flickered at 1 Hz and with 100 ms temporal lag. **(D,E)** The temporal profiles convolved with the TRF by using CRT and OLED. **(F)** Temporal lags shown in CRT and OLED. Black column denotes temporal lag we set for two stimuli; red one denotes temporal lag when stimuli appear on CRT display; green one denotes temporal lag when stimuli appear on OLED display.

DATA SHEET S1 The datasets generated for this study.
