# Supplementary material for: Short-Term Deprivation Does Not Influence Monocular or Dichoptic Temporal Synchrony at Low Temporal Frequency
Source: Front Neurosci. 2020 Apr 28;14:402. doi: 10.3389/fnins.2020.00402 (PMC7198853; doi:10.3389/fnins.2020.00402)
Supplement: FIGURE S1 — The temporal characteristics for the CRT and OLED monitor. (A,B) The temporal response function (TRF) for CRT and OLED. (C) The temporal profiles of the stimuli. The solid and dashed lines represent the two stimuli. Two stimuli flickered at 1 Hz and with 100 ms temporal lag. (D,E) The temporal profiles convolved with the TRF by using CRT and OLED. (F) Temporal lags shown in CRT and OLED. Black column denotes temporal lag we set for two stimuli; red one denotes temporal lag when stimuli appear on CRT display; green one denotes temporal lag when stimuli appear on OLED display. [file Image_1.pdf]

Supplemental Information

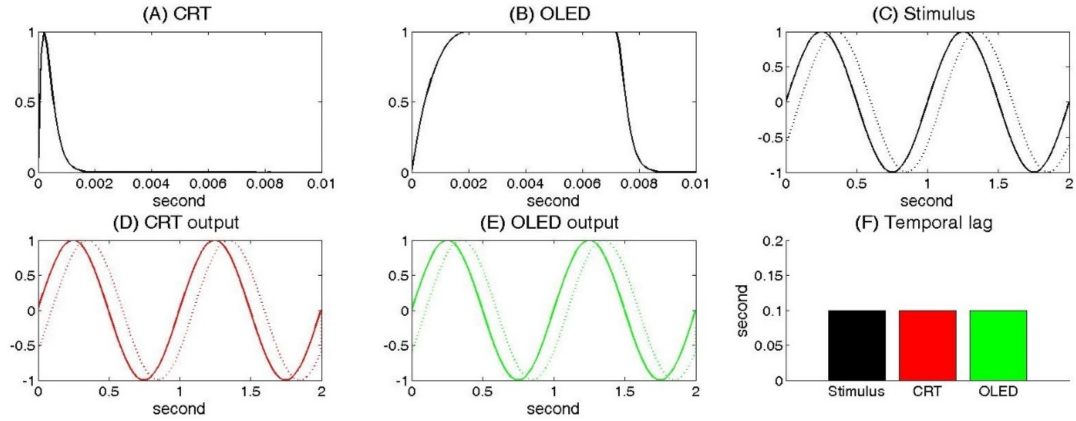

Figure S1. The temporal characteristics for the CRT and OLED monitor.
(A) and (B). The temporal response function (TRF) for CRT and OLED.
(C). The temporal profiles of the stimuli. The solid and dashed lines represent the two stimuli. Two stimuli flickered at 1 Hz and with 100 ms temporal lag.
(D) and (E). The temporal profiles convolved with the TRF by using CRT and OLED.
(F). Temporal lags shown in CRT and OLED. Black column denotes temporal lag we set for two stimuli; red one denotes temporal lag when stimuli appear on CRT display; green one denotes temporal lag when stimuli appear on OLED display.
